# Long‐term results of the Musset surgical technique in the treatment of recto‐vaginal fistulas

**DOI:** 10.1002/ijgo.70532

**Published:** 2025-09-17

**Authors:** Joy Bloomfield, Bassam Haddad, Geoffroy Canlorbe, Cyril Touboul, Edouard Lecarpentier, Yohann Dabi

**Affiliations:** ^1^ Department of Gynecology, Obstetrics and Reproductive Medicine Université de Paris Est Créteil, CHI of Créteil Créteil France; ^2^ Department of Gynecological and Breast Surgery and Oncology, Pitié‐Salpêtrière, Assistance Publique Des Hôpitaux de Paris (AP‐HP) University Hospital Paris France; ^3^ Department of Obstetrics, Gynecology, and Reproductive Medicine, Hôpital Tenon (AP‐HP) Sorbonne University Paris France; ^4^ INSERM U955 Institut Biomédical Henri Mondor Créteil France

**Keywords:** fecal incontinence, Musset, obstetrical fistula, recto‐vaginal fistula, surgical repair

## Abstract

**Objective:**

Recto‐vaginal fistulas (RVF) are a rare pathology, mostly affecting developing countries. They are mainly obstetrical (88% of cases) and have an incidence of 0.5%. Musset's surgical technique in the management of RVF has been the subject of few studies and there is little data in the literature concerning the evaluation of its long‐term efficiency and its morbidity. The aim of our study was to investigate the long‐term results of patients who underwent Musset's surgery for cure of RVF.

**Methods:**

We conducted a monocentric cohort study between January 1, 2002, and December 31, 2020 including patients who had undergone a RVF cure by the Musset technique. These patients were recalled in consultation to be examined and completed a questionnaire designed for this study with validated functional and quality‐of‐life scores. For patients who did not respond to this solicitation, information was retrieved from their postoperative consultations in their medical records. The surgical technique was considered successful if the patient did not present gas or stool incontinence postoperatively.

**Results:**

A total of 38 patients had RVF repair surgery by the Musset technique, and the technique was successful in 48.4% of cases, with high complication and reintervention rates (54.8% and 35.7%, respectively). Half of the patients had a recurrence of symptoms (51.6%) with mainly gas incontinence (43.8%). A total of 11 patients had a long‐term evaluation (28.9%) with a median follow‐up of 5 years (4–15.5 years). Among the patients that were re‐evaluated, all had felt that their life had improved postoperatively and were satisfied with the intervention with a median PGI‐I score of 1 (1–1.5). According to the surveys, quality‐of‐life was improved postoperatively with a median Cleveland score decreasing from 2.5 (1.5–3.75) to 0.5 (0–3.75) and there were no limitations due to physical health or emotional problems according to the SF‐36 questionnaire. Patients had satisfactory sexual intercourses after the procedure with a median high score of 5.2/6 according to the FSFI questionnaire.

**Conclusion:**

The Musset technique provides good functional results and high satisfaction rates and is associated with improved quality‐of‐life postoperatively. However, it is also associated with a high rate of complications and reinterventions. Treatment of RVF can be long and complicated and should therefore be done in referral centers in order to optimize the results.

## 1 INTRODUCTION

Recto‐vaginal fistulas (RVF) are a rare pathology and mainly concern developing countries where they have an incidence of 1.57 per 1000 women in age to procreate.^1^ It is estimated that 88% of RVF are obstetrical^2^ due to the poor access to health care facilities, which is responsible for prolonged labor and dystocia. In a study by Goldaber et al.,^3^ on 24 000 vaginal births, there was an incidence of RVF of 0.5%. The prevalence of obstetric fistulas is difficult to estimate,^4^ but is thought to affect over 2 million women worldwide,^5^ with 50 000 to 100 000 new cases per year in Africa alone.^6^ In Africa, it is estimated that two to three patients suffer from fistulas per 1000 deliveries, with a prevalence of 0.3% in countries where maternal mortality is higher than 500 per 100 000 live births.^7^ The second most common cause of RVF is Crohn's disease, where RVF can occur in up to 10% of patients.^8^ The symptomatology of RVF is variable: they can be paucisymptomatic and therefore be responsible for diagnostic errancy, or they can cause severe symptoms, such as an emission of gas and/or feces through the vagina, responsible for an important alteration in the quality of life of these patients.

Clinical examination is essential in this pathology as it establishes the diagnosis of RVF and allows the RVF to be classified as simple or complex according to the classification of Rothenberger^9^ which orientates the surgical management.

Currently, there is little data concerning the efficiency and outcomes after RVF repair surgery based on the different methods available. Studies analyzing these surgical techniques are often retrospective with a small number of patients included. For example, the studies by Pitel et al.^10^ concerning the Martius flap and by Hull et al.^11^ concerning the gracilis flap in RVF repairs were both retrospective and include only 20 and 22 patients, respectively. Additionally, studies concerning RVF repair surgeries have short‐term median follow‐ups and generally do not evaluate their postoperative long‐term results. In the studies cited above, the median follow‐up was 35 and 7 months, respectively, and none reported their long‐term results.

The Musset technique, initially described in 1971,^12^ is one of the many surgical methods used to treat RVF. This technique has proven to be efficient but has often been studied in combination with other surgical techniques. When studied alone, research concerning this technique has short follow‐up periods with a small number of patients included. In a study of Leroy et al.,^13^ only nine patients having had a RVF repair by the Musset technique were evaluated in order to verify that there was no recurrence of symptoms and the delay of evaluation was 6 to 8 weeks after the operation. In the retrospective study of Chew and Rieger,^14^ seven patients were recalled by a telephone interview with a median follow‐up period of 24 months but none underwent clinical examination in order to assess anatomical and functional outcomes. In a study published in 2001 by Soriano et al.,^15^ 48 patients had a RVF repair by the Musset technique with a follow‐up period between 1 to 3 years postoperatively. Among these patients, five had a reintervention and stool continence was obtained in 98% of patients. Quality of life, mental health, satisfaction rates and sexual function were not evaluated in these patients.

The aim of our study was to study the global outcomes of patients who had undergone repair of their RVF by Musset's technique.

## 2 MATERIALS AND METHODS

### 2.1 Study population

A monocentric cohort study was carried out including patients treated in our tertiary referral center of Intercommunal hospital of Creteil between January 1, 2002, and December 31, 2020. All patients that had undergone a RVF cure by the Musset technique were selected. Patients having had this procedure were identified according to the coding of their surgical procedure. Patients that had a repair of RVF using another technique than Musset and those that did not have a RVF were excluded. Data concerning the included patients were retrieved retrospectively from the medical records.

The committee for the protection of persons gave its approval for this study to be carried out on October 7, 2021 (file no.: 21.00386.000023).

### 2.2 Description of the Musset technique

**FIGURE 1:**
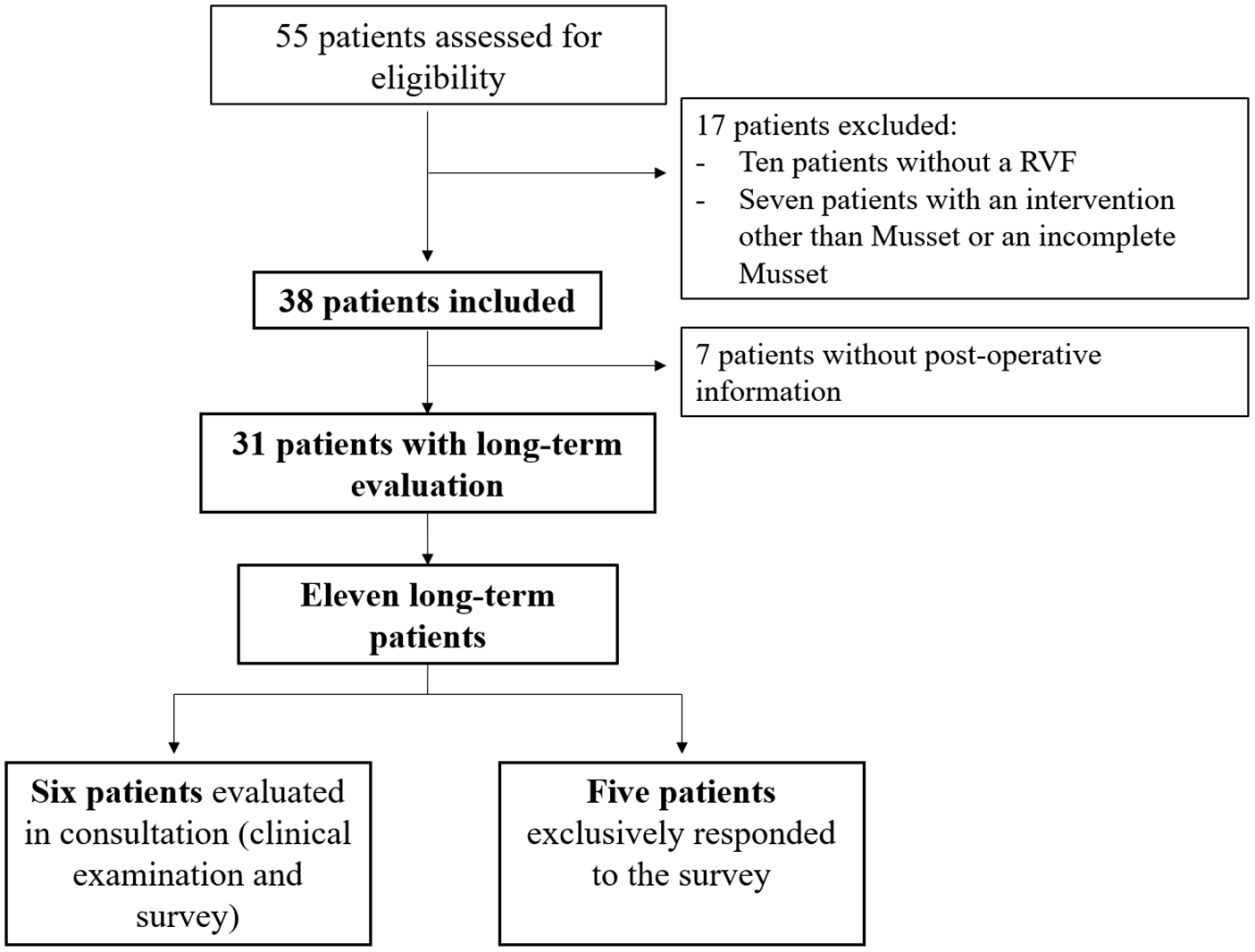
Flow chart.

The first stage is a horizontal incision of the perineum with the removal of the fistula by scalpel and marker threads are placed on the vaginal and rectal mucosal limits. A transverse incision thanks to the exposure of the four marker threads is then made in order to allow the dissection of the rectovaginal plane. The anal canal and sphincter, the vagina and the perineum are then successively closed with absorbable sutures. The technique is visually represented in Figure 1.^16^ This surgical technique can be performed in one or two steps. If performed in two steps, there is a healing period of several months between the first stage and the rest of the operation. Postoperatively, there is daily local care with a hospitalization which lasts 3–4 days and constipation is prevented by laxatives.

### 2.3 Outcome assessment

Patients were recalled for this study using email and telephone calls and had to be evaluated at least 6 months postoperatively. Those answering these solicitations were given a specific survey established for this study and were invited to a consultation for a physical examination. Patients who did not wish to do the consultation were asked to complete the survey only. This survey included questions concerning symptoms, postoperative fertility outcomes, and had validated scores for symptoms of fecal incontinence (Cleveland score), overall satisfaction of the patients (patient global impression of improvement PGI‐I score), quality of life (SF‐36 questionnaire), mental health (WHOQOL‐Bref questionnaire) and sexual life (FSFI questionnaire) (Appendix S1).^17^, ^18^, ^19^, ^20^ The measures realized during the clinical examination were based on the POP‐Q system.^21^

Data concerning the included patients were retrieved in two manners: prospectively for the patients having had a long‐term evaluation (*n* = 11) and retrospectively from the postoperative consultations in the medical records for the patients who did not respond to the solicitations (*n* = 27). The surgical technique was considered successful if the patient was continent for gas and stool at the time of evaluation. Additional analysis on quality of life, mental health, sexual life and anatomical results was done on the recalled patients.

### 2.4 Statistical analysis

Due the small number of patients included the analysis was only descriptive. For quantitative variables, medians and interquartile ranges were calculated. For qualitative variables, number and percentages are reported.

## 3 RESULTS

A total of 38 patients benefited from RVF repair using the Musset technique. Postoperative results were retrieved for 31 patients. Among the included patients, 11 responded to the long‐term follow‐up (*n* = 11/38, 28.9%). Among the 11 recalled patients, six came to the consultation and underwent physical examination.

### 3.1 Results of patients having had a RVF repair by Musset's surgical technique (*n* = 38)

The median age of the patients was 35.5 years (30.3–39.8 years). The majority of the RVFs treated were obstetrical (*n* = 31/38, 81.6%). The perineal status of the 31 patients with obstetrical RVF was: 21 fourth‐degree tears, four third‐degree tears, one tear that was labeled second‐degree, four episiotomies, and one unknown perineal status. A total of 12 patients (*n* = 12/38, 31.6%) had at least one previous procedure for their RVF. Concerning the symptoms that motivated the consultation, 73.6% of the patients had a gas and stool incontinence (*n* = 28/38) (Table 1).

**TABLE 1:** Characteristics of the patients having had a recto‐vaginal fistula repair by Musset's surgical technique (*n* = 38).

Characteristics	Number (*n*)	Percentage (%) IQR
Age	35.5	30.3–39.8
Geographic origin		
Europe	11/22	50
North Africa	2/22	9
Sub‐Saharan Africa	8/22	36.4
Asia	1/22	4.6
Parity	2	1–4
Delivery mode^a^		
Absence of delivery	3/38	7.9
Spontaneous vaginal delivery	37/61	60.7
Instrumental delivery	13/61	21.3
Emergency cesarean section	7/61	11.5
Planned cesarean section	1/61	1.6
Perineum		
Intact	9/49	18.4
First and second‐degree tear	2/49	4.1
Third‐degree tear	4/49	8.2
Fourth‐degree tear	23/49	46.9
Episiotomy	11/49	22.4
Etiology of RVF		
Obstetrical	31/38	81.6
Postoperative	3/38^b^	7.9
Local infection	2/38	5.3
Crohn's disease	1/38	2.6
Local trauma	1/38	2.6
Previous recto‐vaginal repair	12/38	31.6^c^
Number of previous interventions	0	0–1
Type of previous interventions		
Musset technique	6/21	28.6
Fistulotomy	5/21	23.8
Martius flap	4/21	19
Collagen plug	3/21	14.3
Colostomy	2/21	9.5
Seton‐assisted fistula repair	1/21	4.8
Symptoms		
Stool and gas incontinence	28/38	73.6
Gas incontinence	5/38	13.2
Local infection	5/38	13.2
Delay between beginning of incontinence symptoms and consultation (months)	38	12–180

Abbreviations: %, percentage; IQR, interquartile range; med, median; *N*, number; RVF, recto‐vaginal fistula.

^a^ 35/38 patients gave birth with a total of 61 deliveries.

^b^ Posterior colpo‐perineomyorraphy, rectocele repair, removal of the Bartholin gland.

^c^ 12 patients had a previous intervention with a total of 21 interventions.

The RVF was not visualized during clinical examination in 9.4% of cases (*n* = 3/32) and among the RVF visualized, 50% had a diameter >2 cm (*n* = 14/28) (Table 2).

**TABLE 2:** Characteristics of the recto‐vaginal fistulas of the patients having had a recto‐vaginal fistula repair by Musset's surgical technique (*n* = 38).

Characteristics	Number (*n*)	Percentage (%)
RVF visualized during consultation	29/32	90.6
Diameter		
≤2 cm	14/28	50
>2 cm	14/28	50
Localization		
<2/3 lower portion of the vaginal wall	14/29	48.3
≥1/3 upper portion of the vaginal wall	15/29	51.7
Rectal endoscopic ultrasound	17/38	44.7
Visualization of sphincter lesions	16/17	94.1
Other examinations	7/38	18.4
Pelvic MRI	3/38	7.9
Anorectal manometries	3/38	7.9
Anal sphincter electromyographies	2/38	5.3

Abbreviations: %, percentage; MRI, magnetic resonance imaging; *n*, number; RVF, recto‐vaginal fistula.

The majority of the RVF repairs were done during a single procedure (*n* = 24/38, 63.2%). Half of the patients (*n* = 14/31, 45.2%) had no postoperative complications. There was a reintervention in 35.7% of cases (*n* = 5/14): three were Musset techniques and two were fistulotomies (Table 3).

**TABLE 3:** Characteristics of surgical management of the patients having had a recto‐vaginal fistula repair by Musset's surgical technique (*n* = 38).

Characteristics	Number (*n*)	Percentage (%) IQR
Number of Musset technique stages		
1 stage	24/38	63.2
2 stages	14/38	36.8
Duration of hospitalization (days)	4	2.3–5
Postoperative complications (Clavien‐Dindo classification)		
None	14/31	45.2
Grade I	4/31^a^	12.9
Grade II	6/31^b^	19.3
Grade IIIa	1/31^c^	3.2
Grade IIIb	7/31^d^	19.4
Grade IV and V	0/31	0
Reintervention	5/14	35.7
Delay (days)	9	3.5–22.5
Type of reintervention		
Musset technique	3/5	60
Fistulotomy	2/5	40

Abbreviations: %, percentage; IQ, interquartile range; med, median; *n*, number.

^a^ Four scar disunions with local care.

^b^ Six local abscesses with antibiotic therapy.

^c^ Local hematoma with an “X‐stitch” in consultation.

^d^ Seven fistula recurrences.

Half of the patients had a recurrence of their symptoms (*n* = 16/31, 51.6%), with mainly exclusive gas incontinence (*n* = 7/16, 43.8%). Five patients had pregnancies postoperatively (*n* = 5/14, 35.7%) and all deliveries were scheduled cesarean sections (Table 4).

**TABLE 4:** Postoperative characteristics of the patients having had a recto‐vaginal fistula repair by Musset's surgical technique (*n* = 38).

Characteristics	Number (*n*)	Percentage (%) IQR
Recurrence of symptoms	16/31	51.6
Gas incontinence	7/16	43.8
Stool incontinence	3/16	18.7
Gas and stool incontinence	6/16	37.5
Dyspareunia	1/11	9.1
Aesthetic result	21/27	77.8
Subsequent pregnancy^a^	5/14	35.7
Delay surgery—pregnancy (months)	48	28–48
Follow‐up (months)	7	1.5–48

Abbreviations: %, percentage; IQR, interquartile range; med, median; *n*, number.

^a^ All deliveries were scheduled cesarean sections.

### 3.2 Long‐term evaluation of the recalled patients (*n* = 11)

#### Overall satisfaction

Most patients described having a better life postoperatively since the procedure (*n* = 10/11, 90.9%) and all patients were happy with their surgery (*n* = 11/11, 100%) (Table 5). All the patients described diagnostic errancy with the consultation of two to three specialists before the diagnosis of RVF was made.

**TABLE 5:** Results of the surveys in the recalled patients (*n* = 11).

	Patient 1	Patient 2	Patient 3	Patient 4	Patient 5	Patient 6	Patient 7	Patient 8	Patient 9	Patient 10	Patient 11
Delay follow‐up (years)	4	4	11	15	16	19	5	4	17	2	3
Incontinence (Cleveland score)											
Solid stool incontinence											
Preoperative	0	0	3	0	1	1	0	0	0	4	—
Postoperative	0	0	0	0	0	0	0	0	0	0	—
Liquid stool incontinence											
Preoperative	1	3	3	1	1	1	1	3	4	4	—
Postoperative	0	2	1	1	1	1	0	0	0	0	—
Gas incontinence											
Preoperative	4	4	2	1	1	1	1	4	4	4	—
Postoperative	1	0	1	1	1	0	0	3	0	0	—
Pads											
Preoperative	0	3	4	0	1	1	4	3	4	0	—
Postoperative	0	0	4	0	1	1	4	0	0	0	—
Lifestyle alteration											
Preoperative	3	4	4	2	2	3	1	0	0	4	—
Postoperative	1	0	0	3	0	0	4	4	4	0	—
Global satisfaction											
PGI‐I	1	1	1	3	2	1	1	2	1	1	1

*Note*: —, Missing data.

Abbreviation: PGI‐I, patient global impression of improvement.

#### Incontinence (Cleveland score)

Three out of 11 patients (27.3%) had concomitant gas and stool continence. None of the patients had a solid stool incontinence postoperatively, whereas four patients had solid stool incontinence preoperatively (Table 5).

#### Quality of life

In the Cleveland score, quality of life was improved postoperatively and in the SF‐36 score, there were no limitations due to physical health nor due to emotional problems with a median score of 100 (87.50–100) and 100 (66.65–100), respectively.

#### Mental health (WHOQOL‐Bref score)

The median scores were elevated with a median score of 81 (62.50–84.50) regarding physical health, 75 (61–81) regarding psychological health, 75 (62.50–78) regarding social relationships and 75 (66–81) regarding the environment.

#### Sexual function (FSFI score)

All patients who completed the survey had a stable sexual partner. The median scores were 4.2 (3–4.20) regarding desire, 4.2 (3.15–4.95) regarding arousal, 4.8 (4.20–5.25) regarding lubrication, 4.8 (3–5) regarding orgasm, 5.2 (3.40–6) regarding satisfaction and 4.4 (2.60–5.60) regarding pain. The median total score of all the patients was 23.50/36.

#### Physical examination

Six patients were seen in consultation (*n* = 6/11, 54.5%). They had a normal cicatrization of their perineal scars and no fistula was visualized during examination. During the Valsalva maneuvers, there was no emission of gas or stool (Table 6).

**TABLE 6:** Characteristics of the clinical examination during the long‐term evaluation.

	Patient 1	Patient 2	Patient 3	Patient 4	Patient 5	Patient 6
Genital hiatus (cm)	5	3.5	3	3.5	3	4
Perineal body (cm)	2.5	1	2	1.5	4	3
Presence of a perineal gap	Yes	Yes	No	Yes	No	No
Levator ani avulsion	No	Yes	No	No	No	No
Levator ani contraction (/5)	2	2	3	1	4	4
Rectal sensibility	Yes	Yes	Yes	Yes	Yes	Yes
Rectal contraction (/5)	2	1	4	3	5	4

*Note*: Perineal gap: genital hiatus >3 cm and perineal body distance <3 cm and/or natural opening of the genital hiatus to at least one finger during the gynecologic examination, contraction of the levator ani muscles according to the modified Oxford scale according to Laycock; Rectal sensitivity examined during the digital rectal examination.

## 4 DISCUSSION

In our cohort, long term evaluation of Musset's surgical technique showed a success rate of 48.4% in RVF repair, with a rate of complications and reinterventions of 54.8% and 35.7%, respectively. In the patients having a recurrence of incontinence symptoms, it was mostly gas incontinence in 43.8% of cases. All the recalled patients felt a significant improvement in their quality‐of‐life following surgery and were satisfied with their operation. According to the FSFI score, the patients had satisfactory sexual intercourses postoperatively with a median score of 5.2/6 (3.4–6/6).

A study by Göttgens et al.^22^ recently highlighted the poor quality of the published studies concerning RVF repairs. The results are very variable, with closure rates ranging from 0% to over 80%. None of the studies are randomized and a meta‐analysis was not possible. The lack of high‐quality studies on RVF repair could be overcome by establishing expert centers that would allow appropriate multidisciplinary management with experienced surgeons and ensure prolonged follow‐up. Multidisciplinary care is important in the management of RVF since surgery should not only cure the symptoms of incontinence but also help patients to regain a normal self‐image.

The success rate in our study contrasts with that reported by Soriano et al.^15^ Indeed, in their cohort, among the 48 patients that underwent RVF repair by Musset's technique, 98% had a satisfactory functional result and 8.3% patients required iterative surgery. Such discrepancy could be explained by many reasons, including the number of previous interventions, the complexity of the fistulas, the etiology of the fistulas of if the intervention is done is one or two steps. In our study, 81.6% of the fistulas were obstetrical, whereas this etiology represented only 52.1% of the fistulas in the Soriano et al. study^15^ The study by Studniarek et al.^23^ found that obstetrical RVF repairs had lower success rates compared to malignant RVF (43.3% and 68.6%, respectively) and had a significantly increased risk of undergoing more than three procedures. This could be explained by the frequent association of anal sphincter injuries which can be easily overlooked.^24^ However, their preoperative detection may improve postoperative continence.^25^ Performing a rectal endoscopic ultrasound preoperatively is effective in identifying patients with concomitant anal sphincter defects (sensitivity and specificity of 100% for the external sphincter and 100% and 95.5% for the internal sphincter).^26^ In our study, only 17 patients out of the 38 patients benefited from this examination. Another fundamental element to explain such difference could be the prolonged follow‐up that we performed and the difference in criteria for success assessment.

Our success rate could also be explained by the choice of our criteria for success. Indeed, some patients can experience recurrence of incontinence but without a recurrence of the RVF. Factors such as menopause, aging of the sphincter, and decompensation of coping mechanisms can be responsible of delayed incontinence symptoms.^27^ The management of anal incontinence is complex and multifactorial and surgical repair is sometimes insufficient.

Even though incontinence symptoms persisted in the 11 patients that were recalled, they were all satisfied with their intervention and their quality of life. Indeed, concerning the satisfaction rates of our study, 10 patients described having a better life postoperatively since the procedure and all answered that they were happy with their surgery. Concerning the quality of life, it was improved postoperatively according to the Cleveland scale with a median score decreasing from 2.5 (1.5–3.75) to 0.5 (0–3.75) and there were no limitations due to physical health nor due to emotional problems according to the SF‐36 questionnaire with a median score of 100 (87.50–100) and 100 (66.65–100), respectively. The high satisfaction rate of our study could be explained by CHI Creteil's historical involvement in fistula repair and in vulvar and perineal surgeries, making it a reference center in the matter. Indeed, many patients have been operated on by Paniel et al., a leading expert in vulvar pathologies.^28^, ^29^ These results are consistent with the study of Leroy et al.^13^ who also recalled patients who had undergone a RVF repair by the Musset technique: three of the four patients were satisfied with their procedure, had a stable quality of life and an absence of physical and social repercussions even though a certain degree of incontinence persisted.

The sexual function score of patients having had a RVF repair was higher in our study compared to previous studies. In the study by El‐Gazzaz et al.,^30^ half of the patients that were recalled had regained their sexual activity postoperatively and 25.5% had dyspareunia.

The study by Studniarek et al.^23^ analyzed the success rate of up to 20 different surgical techniques in the repair of RVF, with an overall success rate of 37.3%. Among the procedures with the highest chance of success was 57.9% for the endorectal flap (22/38 patients), 55.2% for abdominal resections with and without proximal bypass (16/29) and 53.1% for the Musset technique (17/32). The choice of the Musset technique in RVF repairs could be based on the etiology of the fistula and could be well adapted to obstetrical fistulas since it allows a repair of the anal sphincter in the same operative time.

In the present study, some limitations are worth underlining. First, the long delay between evaluation and the time of surgery could have induced a risk of bias including recall bias with patients over‐evaluating their discomfort prior to surgery. However, a large period of inclusion was required due to the rarity of the procedure and to achieve long follow‐up. The monocentric inclusion in our tertiary referral center induces a risk of selection bias as more severe patients were referred for management, thus underestimating the benefit of this surgical technique. Indeed, in our cohort, 12 patients had undergone an attempt of RVF repair prior to the index surgery. Second, the limited number of patients, due to the rarity of this condition in occidental countries, did not permit a comparison between the incontinent and continent patients. Furthermore, 27 patients were lost to follow‐up which could be explained by the traumatic experience for these patients of having a RVF. During the visit, a patient described how traumatic this experience had been for her and how difficult it was to discuss her illness. Third, the scores used could bias the evaluation of the surgical impact. The scores used are usually based on symptoms preceding 4 weeks from the moment the patient took the test. The results of the SF‐36 and WHOQOL‐Bref scores were strongly biased with decreased results in a patient who had recently had a fetal death in utero before the study. Life events can therefore modify the scoring and are more representative of quality of life and mental health in general than post‐RVF treatment. Similarly concerning the FSFI score: two of the recalled patients had not had recent sexual relations and were therefore unable to answer all the questions. Eventually, the different questionnaires were not available prior surgery to definitely assess the state of all patients at the time they required/decided to undergo surgery.

## 5 CONCLUSION

The Musset technique provides good functional results with a success rate of 51.6% and a reduction in the intensity of incontinence symptoms. There were excellent satisfaction rates and an improved quality‐of‐life postoperatively. However, this repair technique is associated with a high rate of complications and reinterventions, resulting in long‐term care which should be done in referral centers in order to optimize the chances of success.

## AUTHOR CONTRIBUTIONS

Planning the study—Organization—Study administration: BH, YD, GC, CT. Data collection and analysis: JB, EL, BH, CT, YD. Drafting first version of the manuscript: JB, EL, YD. Revising manuscript for critical intellectual content: all authors.

## CONFLICT OF INTEREST STATEMENT

The authors declare that they do not have any conflicts of interest.

## Supporting information


Appendix S1.

## Data Availability

Research data are not shared.

## References

[1] Adler AJ , Ronsmans C , Calvert C , Filippi V . Estimating the prevalence of obstetric fistula: a systematic review and meta‐analysis. BMC Pregnancy Childbirth. 2013;13(1):246. 24373152 10.1186/1471-2393-13-246 PMC3937166

[2] Champagne BJ , McGee MF . Rectovaginal Fistula. Surg Clin North Am. 2010;90(1):69‐82. 20109633 10.1016/j.suc.2009.09.003

[3] Goldaber KG , Wendel PJ , McIntire DD , Wendel GD . Postpartum perineal morbidity after fourth‐degree perineal repair. Am J Obstet Gynecol. 1993;168(2):489‐493. 8438915 10.1016/0002-9378(93)90478-2

[4] Cowgill KD , Bishop J , Norgaard AK , Rubens CE , Gravett MG . Obstetric fistula in low‐resource countries: an under‐valued and under‐studied problem—systematic review of its incidence, prevalence, and association with stillbirth. BMC Pregnancy Childbirth. 2015;15(1):193. 26306705 10.1186/s12884-015-0592-2 PMC4550077

[5] Murray CJL , Lopez AD , Organization WH . Health Dimensions of Sex and Reproduction: the Global Burden of Sexually Transmitted Diseases, HIV, Maternal Conditions, Perinatal Disorders, and Congenital Anomalies. Boston: Harvard School of Public Health; 1998. Accessed Jul 6, 2021. https://apps.who.int/iris/handle/10665/42161

[6] Bacon C , United Nations Population Fund , EngenderHealth (Firm) . Obstetric fistula needs assessment report: findings from nine African countries. UNFPA: EngenderHealth; 2003.

[7] United Nations Population Fund . Second Meeting of the Working Group for the Prevention and Treatment of Obstetric Fistula. Accessed May 4, 2022. https://www.unfpa.org/publications/second‐meeting‐working‐group‐prevention‐and‐treatment‐obstetric‐fistula

[8] Hannaway CD , Hull TL . Current considerations in the management of rectovaginal fistula from Crohn's disease. Color Dis. 2008;10(8):747‐755. 10.1111/j.1463-1318.2008.01552.x 18462243

[9] Rothenberger DA , Goldberg SM . The management of rectovaginal fistulae. Surg Clin North Am. 1983;63(1):61‐79. 6338609 10.1016/s0039-6109(16)42930-0

[10] Pitel S , Lefevre JH , Parc Y , Chafai N , Shields C , Tiret E . Martius advancement flap for low rectovaginal fistula: short‐ and long‐term results. Colorectal Dis. 2011;13(6):e112‐e115. 21564462 10.1111/j.1463-1318.2011.02544.x

[11] Hull TL , Sapci I , Lightner AL . Gracilis flap repair for Reoperative rectovaginal fistula. Dis Colon Rectum. 2023;66(1):113‐117. 34759248 10.1097/DCR.0000000000002249

[12] Musset R . Surgical treatment of rectovaginal fistula of the lower two‐thirds of the vagina. Lyon Chir. 1971;67(1):68‐70. 5576796

[13] Leroy A , Azaïs H , Giraudet G , Cosson M . Quality of life and symptoms before and after surgical treatment of rectovaginal fistula. Prog Urol. 2017;27(4):229‐237. 28065390 10.1016/j.purol.2016.12.001

[14] Chew SSB , Rieger NA . Transperineal repair of obstetric‐related anovaginal fistula. Aust N Z J Obstet Gynaecol. 2004;44(1):68‐71. 15089873 10.1111/j.1479-828X.2004.00175.x

[15] Soriano D , Lemoine A , Laplace C , et al. Results of recto‐vaginal fistula repair: retrospective analysis of 48 cases. Eur J Obstet Gynecol Reprod Biol. 2001;96(1):75‐79. 11311765 10.1016/s0301-2115(00)00411-5

[16] Cosson M , Lucot JP , Rubod C , Collinet P , Vinatier D . Cure de la fistule obstétricale rectovaginale en un temps selon Musset. J Gynecol Obstet Biol Reprod. 2005;34(5):513.

[17] Srikrishna S , Robinson D , Cardozo L . Validation of the patient global impression of improvement (PGI‐I) for urogenital prolapse. Int Urogynecol J. 2010;21(5):523‐528. 20013110 10.1007/s00192-009-1069-5

[18] Leplège A , Ecosse E , Verdier A , Perneger TV . The French SF‐36 health survey: translation, cultural adaptation and preliminary psychometric evaluation. J Clin Epidemiol. 1998;51(11):1013‐1023. 9817119 10.1016/s0895-4356(98)00093-6

[19] Baumann C , Erpelding ML , Régat S , Collin JF , Briançon S . The WHOQOL‐BREF questionnaire: French adult population norms for the physical health, psychological health and social relationship dimensions. Rev Epidemiol Sante Publique. 2010;58(1):33‐39. 20096515 10.1016/j.respe.2009.10.009

[20] Rosen R , Brown C , Heiman J , et al. The female sexual function index (FSFI): a multidimensional self‐report instrument for the assessment of female sexual function. J Sex Marital Ther. 2000;26(2):191‐208. 10782451 10.1080/009262300278597

[21] Persu C , Chapple C , Cauni V , Gutue S , Geavlete P . Pelvic organ prolapse quantification system (POP–Q)—a new era in pelvic prolapse staging. J Med Life. 2011;4(1):75‐81. 21505577 PMC3056425

[22] Göttgens KW , Smeets RR , Stassen LP , Beets G , Breukink SO . The disappointing quality of published studies on operative techniques for rectovaginal fistulas: a blueprint for a prospective multi‐institutional study. Dis Colon Rectum. 2014;57(7):888‐898. 24901691 10.1097/DCR.0000000000000147

[23] Studniarek A , Abcarian A , Pan J , Wang H , Gantt G , Abcarian H . What is the best method of rectovaginal fistula repair? A 25‐year single‐center experience. Tech Coloproctol. 2021;25(9):1037‐1044. 34101044 10.1007/s10151-021-02475-y

[24] Khanduja KS , Yamashita HJ , Wise WE , Aguilar PS , Hartmann RF . Delayed repair of obstetric injuries of the anorectum and vagina. A stratified surgical approach. Dis Colon Rectum. 1994;37(4):344‐349. 8168413 10.1007/BF02053595

[25] Tsang CB , Madoff RD , Wong WD , et al. Anal sphincter integrity and function influences outcome in rectovaginal fistula repair. Dis Colon Rectum. 1998;41(9):1141‐1146. 9749498 10.1007/BF02239436

[26] Deen KI , Kumar D , Williams JG , Olliff J , Keighley MR . Anal sphincter defects. Correlation between endoanal ultrasound and surgery. Ann Surg. 1993;218(2):201‐205. 8343001 10.1097/00000658-199308000-00013 PMC1242931

[27] Saldana Ruiz N , Kaiser AM . Fecal incontinence—challenges and solutions. World J Gastroenterol. 2017;23(1):11‐24. 28104977 10.3748/wjg.v23.i1.11 PMC5221273

[28] Paniel BJ , Truc JB , de Margerie V , Chantraine J , Poitout P . Vulvo‐perineal surgery. J Gynecol Obstet Biol Reprod (Paris). 1984;13(1):91‐100. 6725881

[29] Paniel BJ , Truc JB , Beuzit JM , Pelissier C , Poitout P . Diagnosis of congenital abnormalities of the vulva and vagina. Ann Pediatr (Paris). 1987;34(1):11‐25. 3827104

[30] El‐Gazzaz G , Hull TL , Mignanelli E , Hammel J , Gurland B , Zutshi M . Obstetric and Cryptoglandular rectovaginal fistulas: long‐term surgical outcome; quality of life; and sexual function. J Gastrointest Surg. 2010;14(11):1758‐1763. 20593308 10.1007/s11605-010-1259-y

