# Supplementary material for: Long‐term results of the Musset surgical technique in the treatment of recto‐vaginal fistulas
Source: Int J Gynaecol Obstet. 2025 Sep 17;172(3):1656–64. doi: 10.1002/ijgo.70532 (PMC12936632; doi:10.1002/ijgo.70532)
Supplement: Supplementary file 1 — Appendix S1. [file IJGO-172-1656-s001.docx]

**Supplementary figure 1 – Musset’s surgical technique**


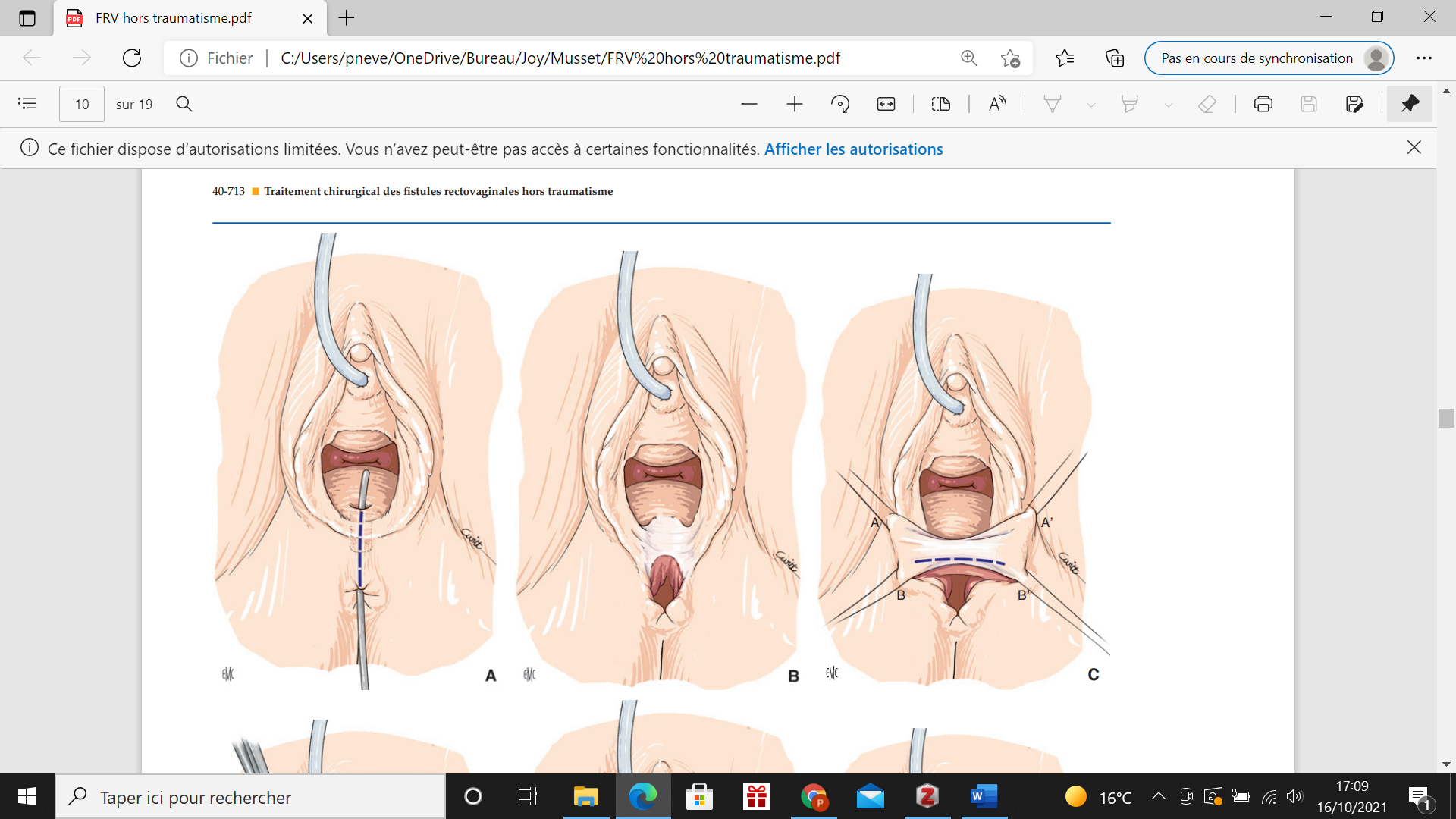


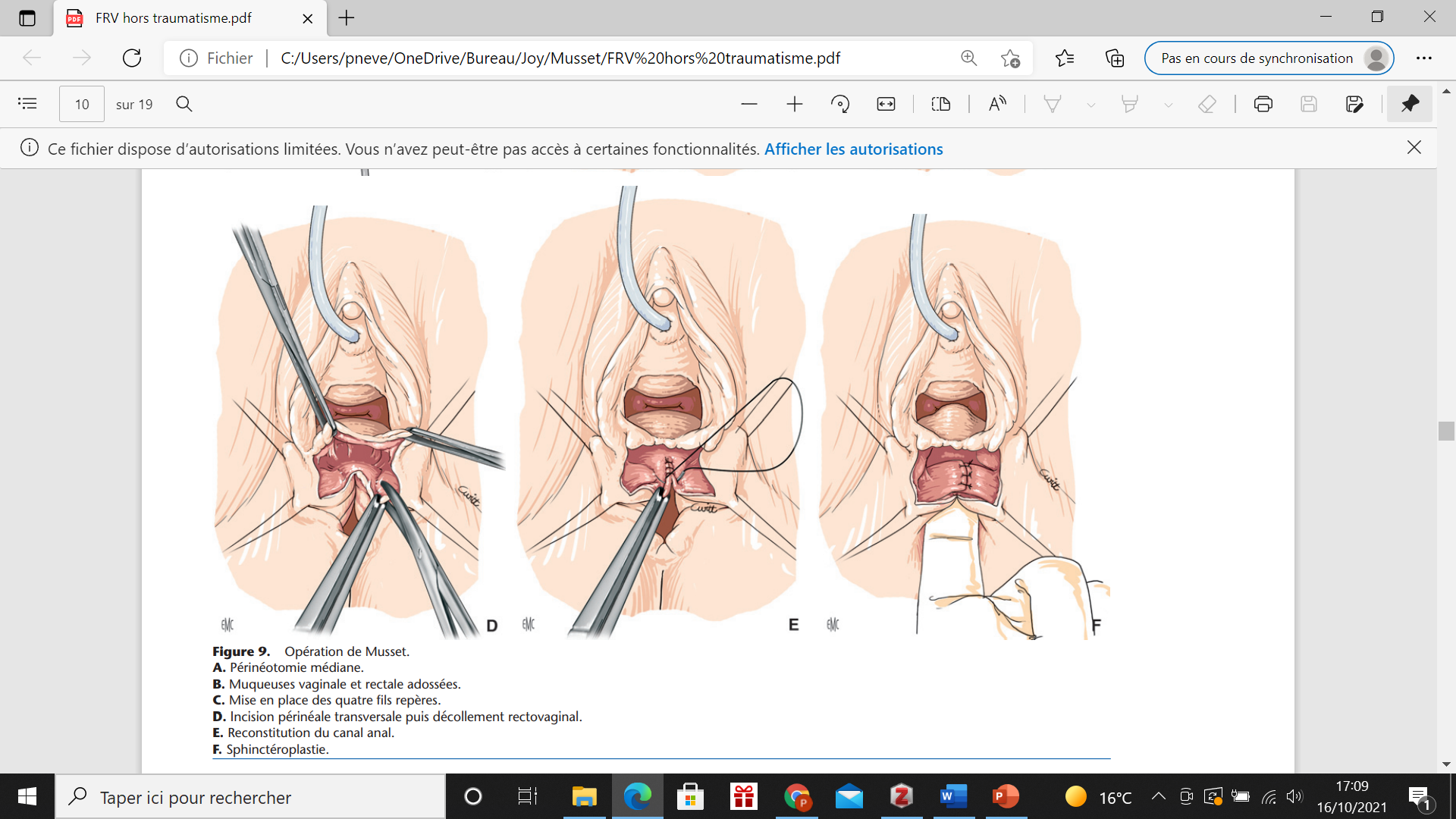


*A. Removal of the RVF by a median incision of the perineum. This consists in the first phase of the Musset surgical technique. If there is a local inflammation, there is a delay of a few weeks after this step in order to allow a cicatrization of the incision.*

*B. Cicatrization of the vaginal and rectal mucosa (if the Musset is performed in 2 stages).*

*C. Placement of four marker threads. If the Musset is performed in two stages, the threads put in tension the perineal scar. If the Musset is performed in one step, the threads are placed on the vaginal and rectal cutaneous-mucosal limits (2 on the right and 2 on the left).*

*D. Transverse perineal incision then recto-vaginal separation.*

*E. Reconstruction of the anal canal with simple stiches with resorbable threads (Vicryl 1/0).*

*F. Sphincteroplasty by reconstructing the external anal sphincter by “U” stiches with resorbable threads (Vicryl 1/0).*

*The vaginal and perineal incisions are then sutures with simple stitches (resorbable threads of Vicryl 1/0 and then Vicryl rapide 2/0) (10).*

**Supplementary figure 2 - Survey**


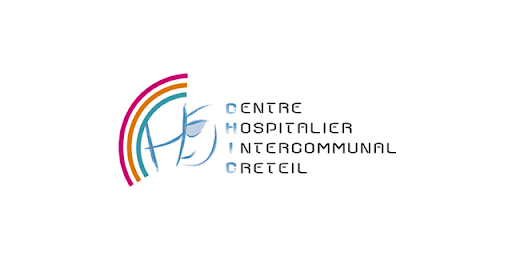

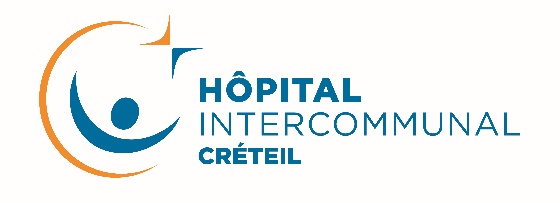


**POST-OPERATIVE EVALUATION AFTER MUSSET SURGICAL TECHNIQUE**

1. **FOLLOW-UP :**

| 1. **a. RECURRENCE OF SYMPTOMS : YES □ NO □**   If yes, please specify :   - Fecal incontinence **□** - Gaz incontinence **□** - Both **□** - Vaginal and/or rectal discharge **□**   **1. b . DID YOU HAVE ANY COMPLICATIONS FOLLOWING THE PROCEDURE? : YES □ NO □**  If yes, specify the complication and location:   - Complication : - Location :   **1. c. DID YOU REQUIRE A FISTULA-RELATED HOSPITALIZATION? : YES □ NO □**  If yes, specify reason and location:   - Reason for hospitalization: - Location :  1. **d. REINTERVENTION : YES □ NO □**   If yes, specify year, location and type of intervention:  - Year of reintervention :  - Location :  - Type of intervention :  **1. e. CONCERNING YOUR PREGNANCIES (give details for each pregnancy):**  Pregnancy #1:  - Type of delivery: vaginal delivery □ cesarean section □  - Year:  - Maternity:  - Weight (in g):  - Perineal tears: YES □ NO □  - Episiotomy: YES □ NO □  Pregnancy #2:  - Type of delivery: vaginal delivery □ cesarean section □  - Year:  - Maternity:  - Weight (in g):  - Perineal tears: YES □ NO □  - Episiotomy: YES □ NO □  Pregnancy #3:  - Type of delivery: vaginal delivery □ cesarean section □  - Year:  - Maternity:  - Weight (in g):  - Perineal tears: YES □ NO □  - Episiotomy: YES □ NO □  Pregnancy #4:  - Type of delivery: vaginal delivery □ cesarean section □  - Year:  - Maternity:  - Weight (in g):  - Perineal tears: YES □ NO □  - Episiotomy: YES □ NO □   1. **f. PREGNANCY AFTER YOUR FISTULA OPERATION : YES □ NO □**   **1. g. HAVE YOU USED PERINEAL REHABILITATION FOR YOUR SYMPTOMS?:**  **YES □ NO □** |
| --- |

**2 SYMPTOMS :**

| 1. **a. HAS YOUR LIFE IMPROVED SINCE THE OPERATION? YES □ NO □** 2. **b. ESTHETIC FINISH: SATISFIED □ NOT SATISFIED □** 3. **c. PAIN DURING INTERCOURSE: YES □ NO □** 4. **d. CONCERNING GAZ AND/OR FECAL INCONTINENCE :**  - **BEFORE YOUR FISTULA OPERATION :**   **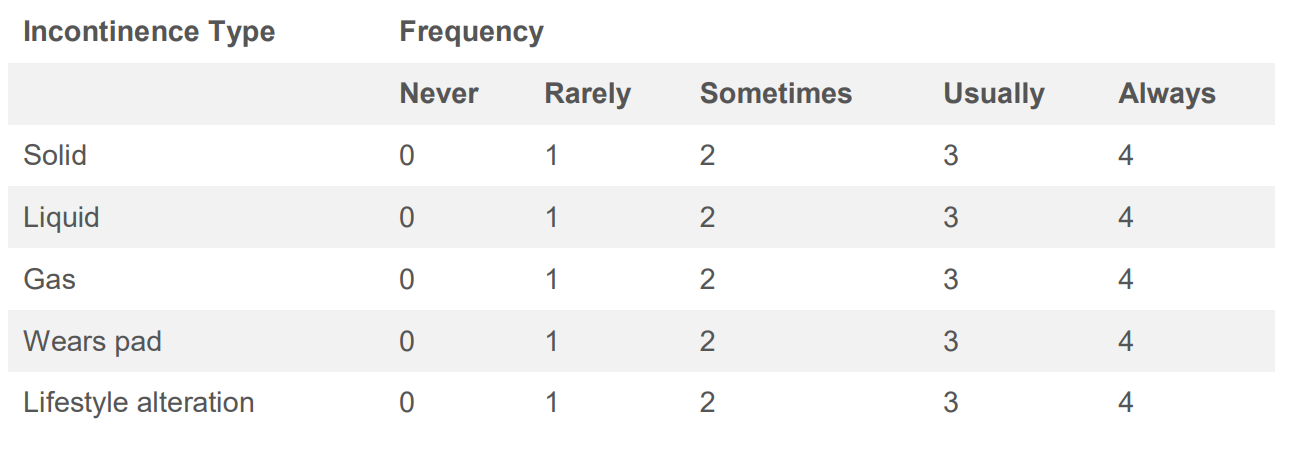**  **• Rarely : 1/month • Sometimes : 1/month • Usually : 1/week • Always : >1/day.**   - **AFTER YOUR FISTULA OPERATION :**   **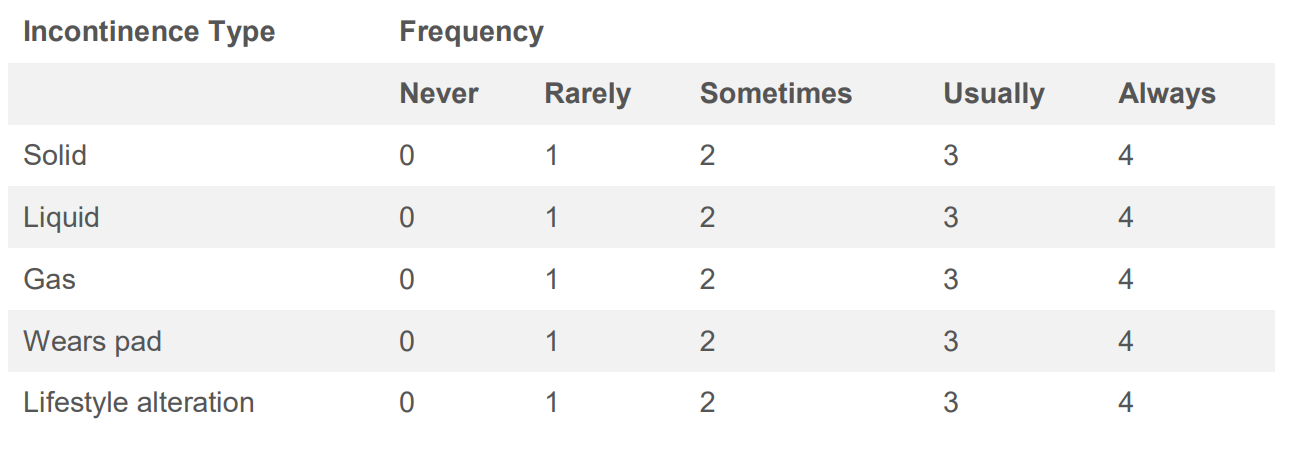**  **• Rarely : 1/month • Sometimes : 1/month • Usually : 1/week • Always : >1/day.** |
| --- |

**3. OVERALL SATISFACTION**

**3. a. ARE YOU HAPPY TO HAVE HAD THE OPERATION? YES □ NO □**

**3. b. PATIENT GLOBAL IMPRESSION AND IMPROVEMENT (PGI-I)**

Circle the number that best describes how your problems are now, compared with how they were before treatment.


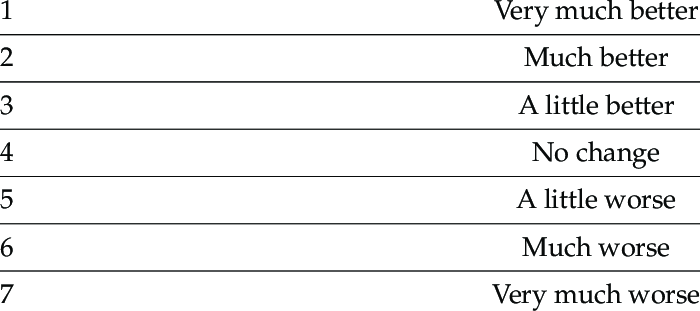


1. **QUALITY OF LIFE (SF 36) :**


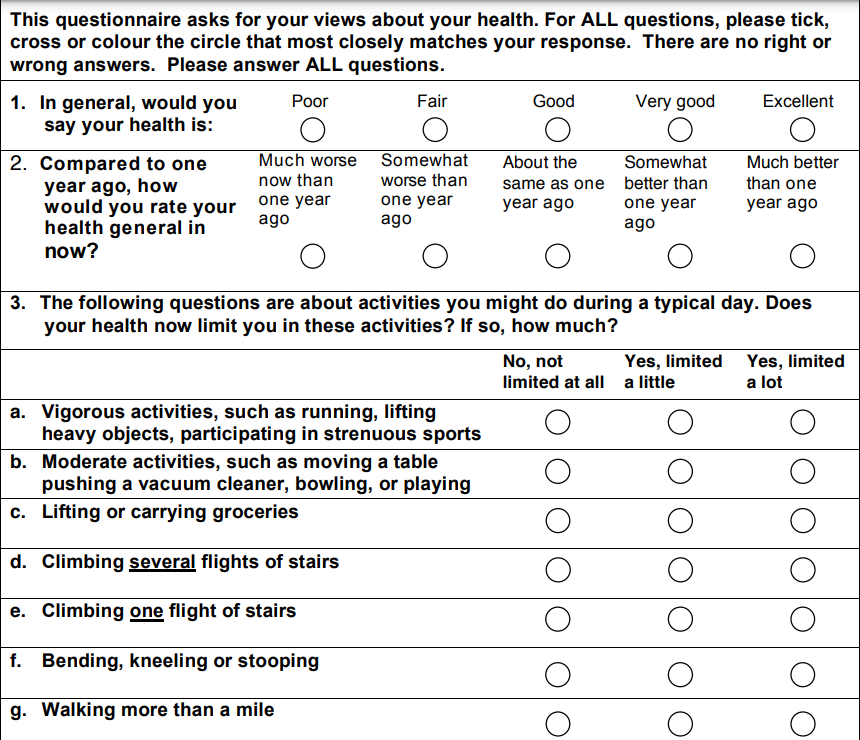


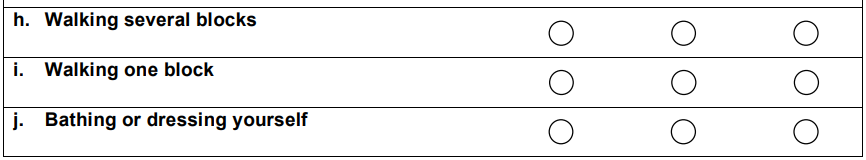


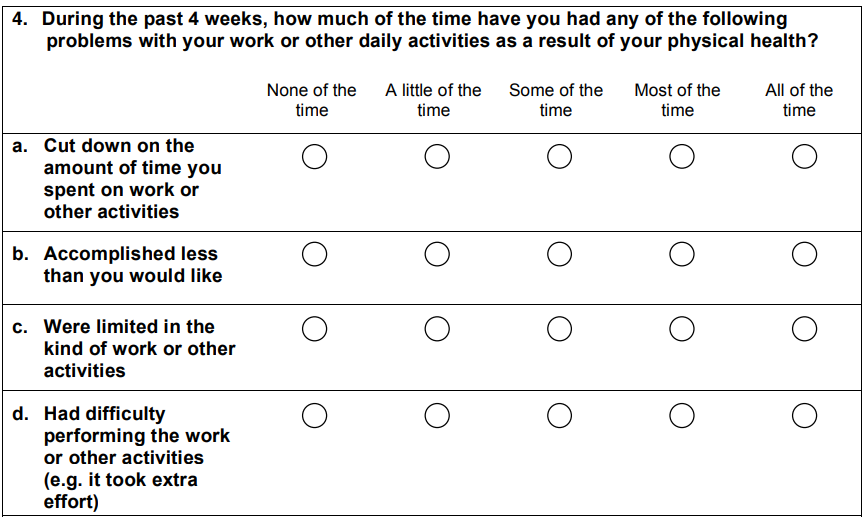


**
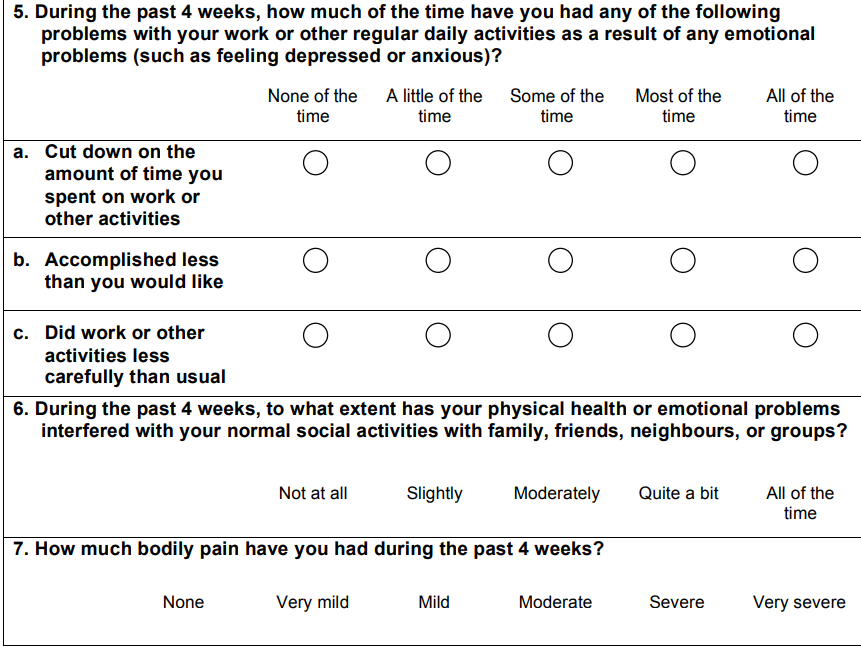
**

**
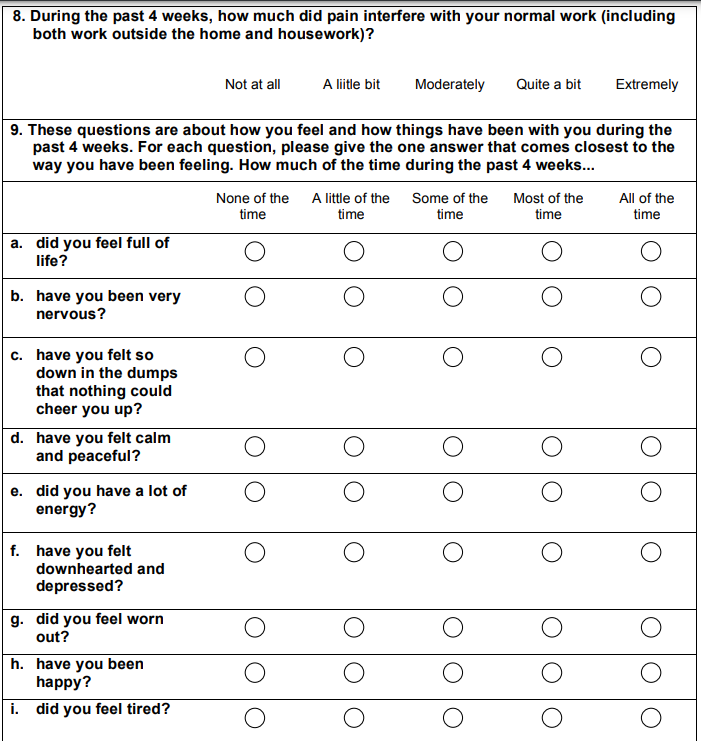
**

**
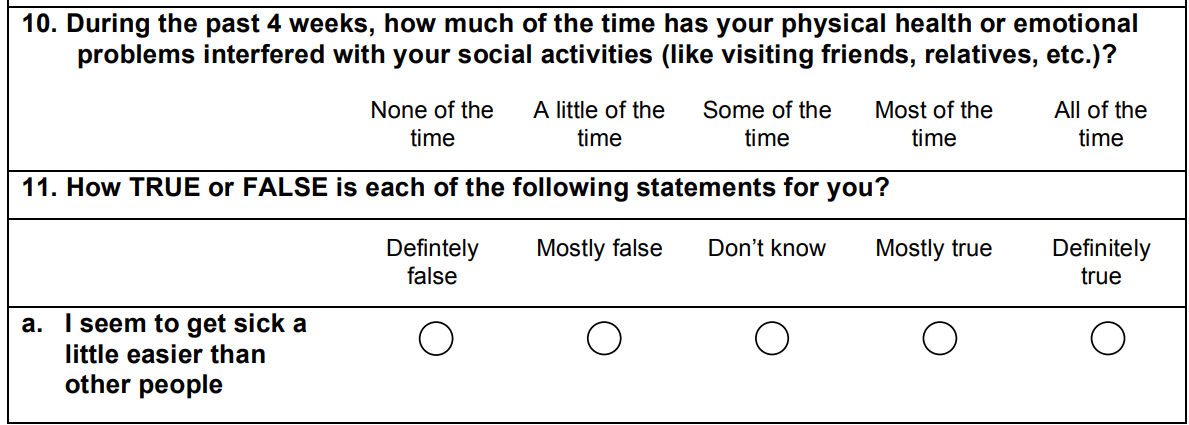
**

**
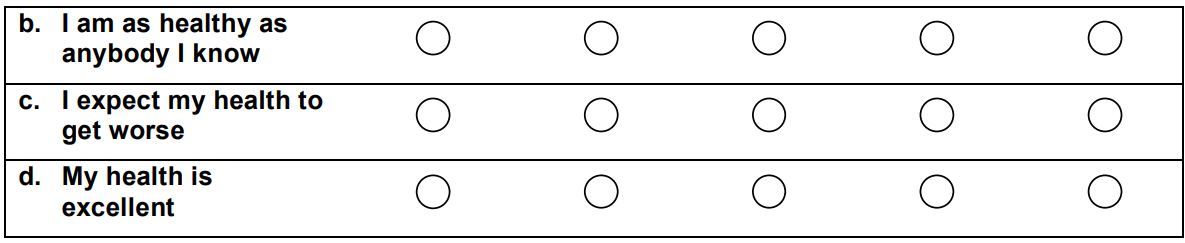
**

1. **MENTAL HEALTH (WHOQOL – Bref) :**


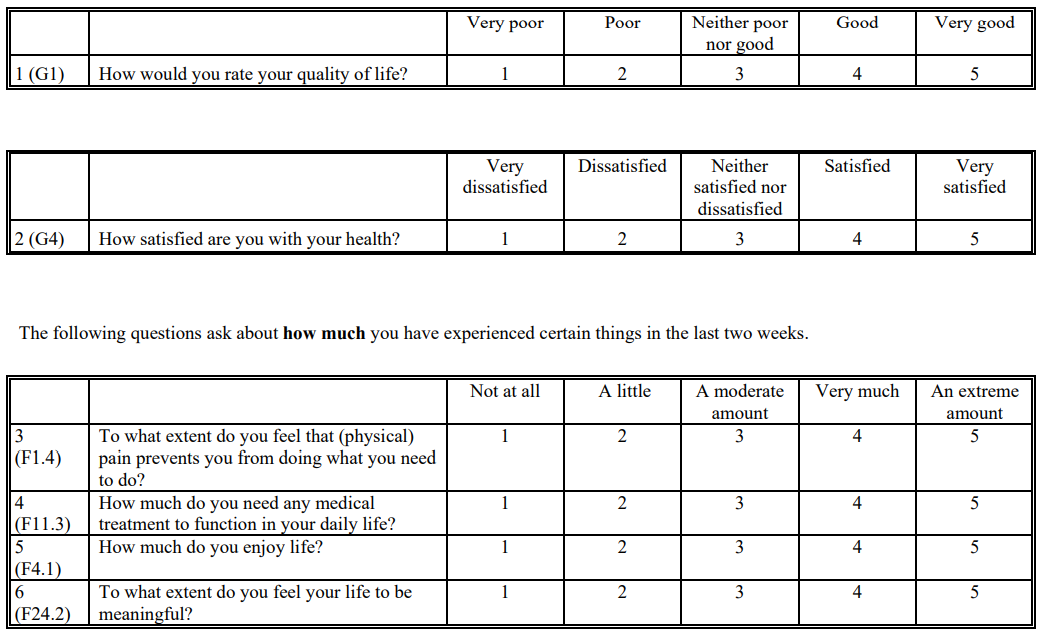


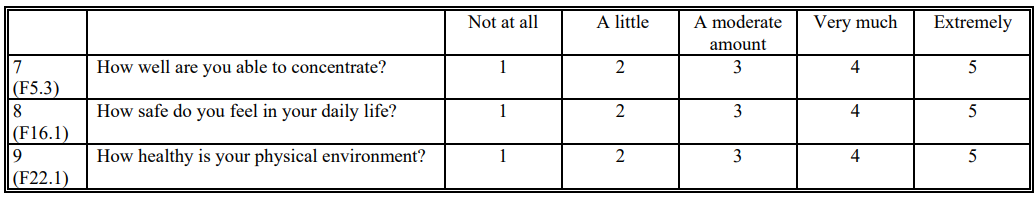


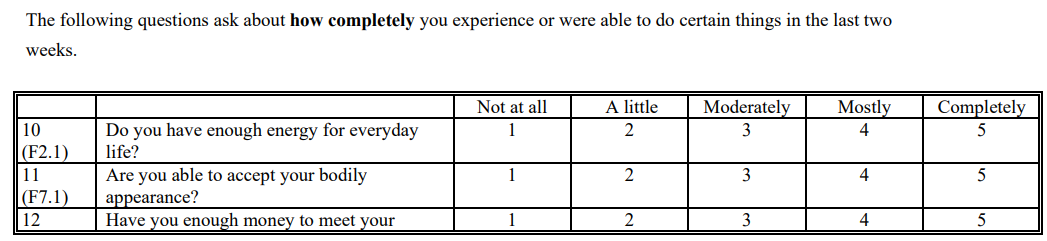


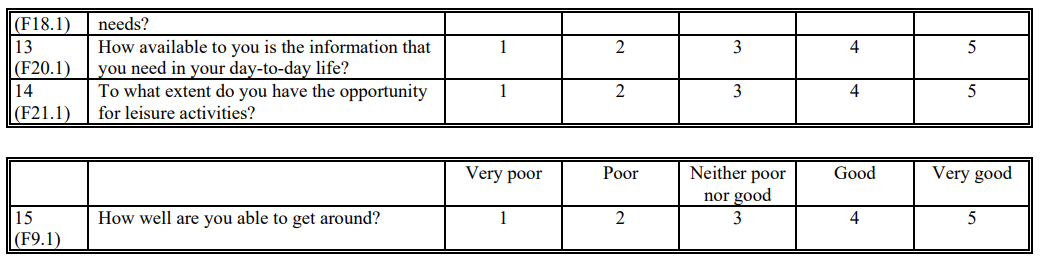


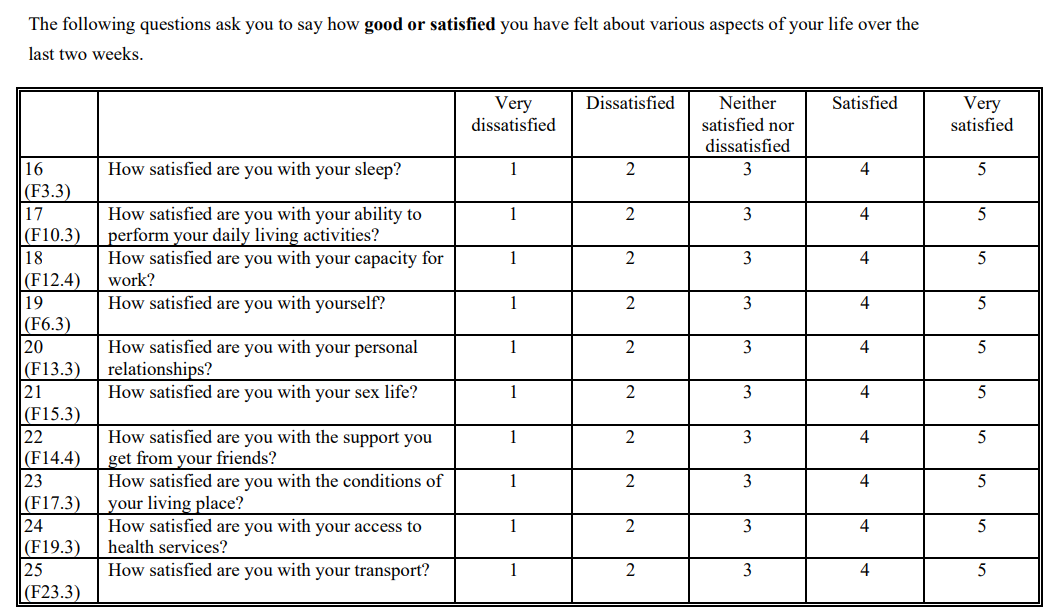


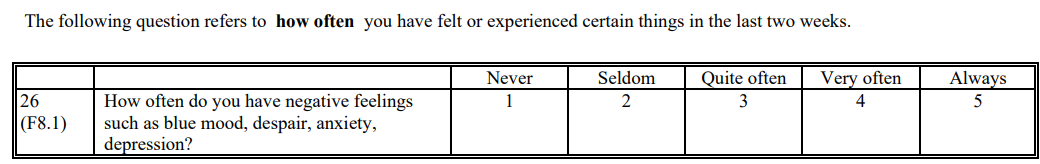


1. **SEXUAL FUNCTION (FSFI) :**


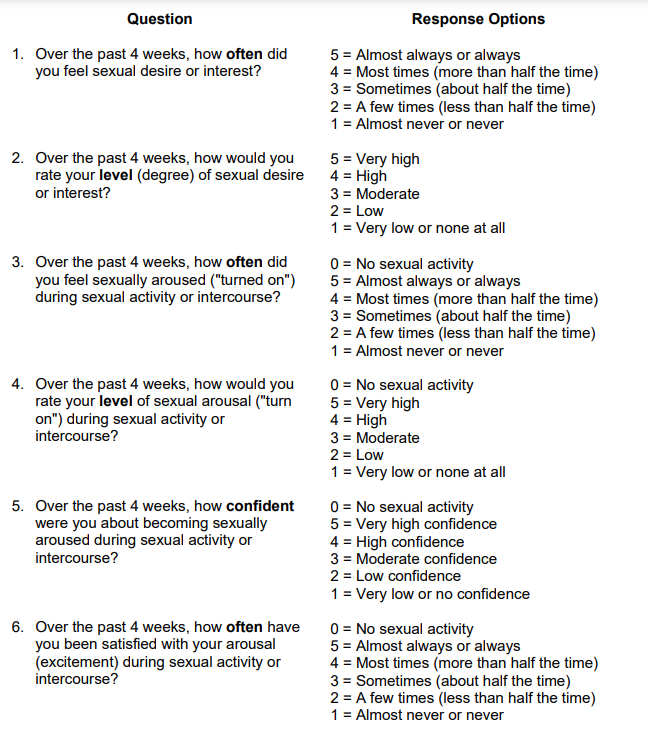


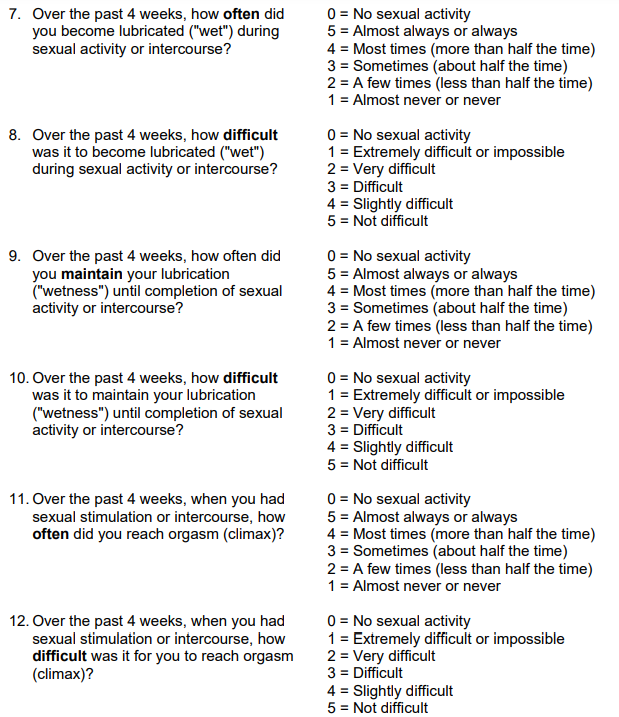


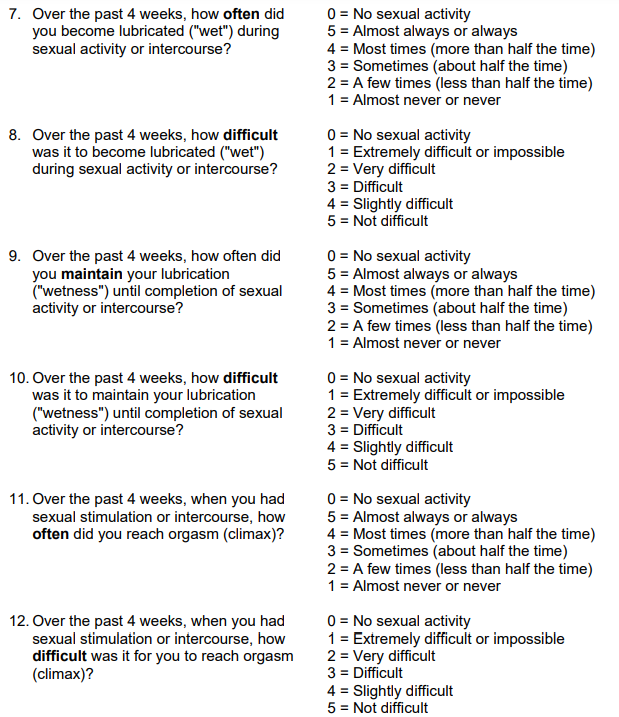


**
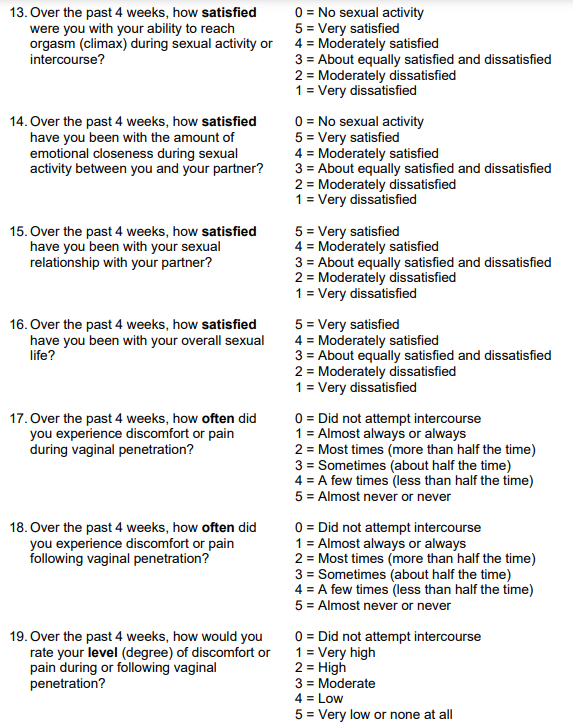
**

**Supplementary table 1 - Characteristics of the 11 recalled patients**

|  | | **Patient 1** | **Patient 2** | **Patient 3** | **Patient 4** | **Patient 5** | **Patient 6** | **Patient 7** | **Patient 8** | **Patient 9** | **Patient 10** | **Patient 11** |
| --- | --- | --- | --- | --- | --- | --- | --- | --- | --- | --- | --- | --- |
| **Age during operation** | | 39 | 35 | 40 | 34 | 36 | 37 | 22 | 17 | 30 | 36 | 24 |
| **Number of previous interventions** | | 0 | 1* | 1* | 0 | 0 | 0 | 0 | 0 | 1** | 6* | 0 |
| **Localization RVF** | | 2/3 lower portion of the vaginal wall | 1/3 upper portion of the vaginal wall | - | 1/3 upper portion of the vaginal wall | 1/3 upper portion of the vaginal wall | 2/3 lower portion of the vaginal wall | - | - | - | 1/3 upper portion of the vaginal wall | 2/3 lower portion of the vaginal wall |
| **Diameter RVF** | | ≤ 2 cm | > 2 cm | - | > 2cm | > 2 cm | ≤ 2 cm | - | - | - | > 2 cm | ≤ 2 cm |
| **Year of intervention** | | 2018 | 2018 | 2011 | 2007 | 2006 | 2003 | 2017 | 2018 | 2005 | 2020 | 2019 |
| **Etiology of RVF** | | Obstetrical | Obstetrical | Obstetrical | Obstetrical | Obstetrical | Obstetrical | Obstetrical | Trauma | Obstetrical | Local infection | Obstetrical |
| **Post-operative complication (Clavien-Dindo classification)** | | Yes (1) | - | No | Yes (1) | Yes (1) | No | No | No | Yes (1) | Yes (3b) | No |
|  | **Detail complication** | Local abscess | - |  | Local abscess | Local abscess |  |  |  | Local abscess | Scar disunion |  |
| **Reintervention** | | No | No | - | No | No | No | No | No | No | No | Yes (Musset) |
| **Ulterior pregnancies (delay in months)** | | No | No | No | No | Yes (48) | No | Yes (48) | Yes (28) | Yes (24) | No | No |

* unknown, ** fistulotomy, RVF: rectovaginal fistula, - missing data
